# Supplementary material for: SyNDI: synchronous network data integration framework
Source: BMC Bioinformatics. 2018 Nov 6;19:403. doi: 10.1186/s12859-018-2426-5 (PMC6219086; doi:10.1186/s12859-018-2426-5)
Supplement: Supplementary file 5 — Files of the example presented in Additional file 4. This additional file is a zip package that contains all files used in Additional file 4. Detailed descriptions of each of these files are presented in a README file included in this zip package. (GZ 30411 kb) [file 12859_2018_2426_MOESM5_ESM.gz › SyNDITools/Meme2Fimo/meme_4.9.0_4.tar.gz!/meme_4.9.0/website/html/meme-intro.html]

MEME - Overview


|  |  |
| --- | --- |
|  | MEME Suite The MEME Suite web application requires the use of JavaScript   Javascript doesn't seem to be available on your browser.  Overview  MEME is a tool for discovering **motifs** in a group of related DNA or protein sequences.  A motif is a sequence pattern that occurs repeatedly in a group of related protein or DNA sequences. MEME represents motifs as position-dependent letter-probability matrices which describe the probability of each possible letter at each position in the pattern. Individual MEME motifs do not contain gaps. Patterns with variable-length gaps are split by MEME into two or more separate motifs.  MEME takes as input a group of DNA or protein sequences and outputs as many motifs as requested. MEME uses statistical modeling techniques to automatically choose the best width, number of occurrences, and description for each motif.  Your MEME results consist of:   - your  **MEME results in**     HTML format - your  **MEME results in**     XML format - your  **MEME results in**     TEXT format - and the    **MAST results** of   searching your input sequences for the motifs found by MEME using   **MAST**. |
